# Assessing the Impact of Family Caregivers in Clinical Encounter Participation on Patient Activation in the Metastatic Breast Cancer Setting

**DOI:** 10.21203/rs.3.rs-3677175/v1

**Published:** 2023-12-04

**Authors:** Nicole L. Henderson, Tanvi Padalkar, Garrett Bourne, Emma K. Hendrix, Courtney P. Williams, J. Nicholas Odom, Kristen Triebel, Gabrielle Rocque

**Affiliations:** University of Alabama at Birmingham; University of Alabama at Birmingham; University of Alabama at Birmingham; University of Alabama at Birmingham; University of Alabama at Birmingham; University of Alabama at Birmingham; University of Alabama at Birmingham; University of Alabama at Birmingham

**Keywords:** Cancer, Oncology, Family Caregivers, Decision-Making, Patient Activation, Treatment planning, Satisfaction with care, Caregiving

## Abstract

**Objective:**

Caregivers support individuals undergoing cancer treatment by assisting with activities, managing care, navigating healthcare systems, and communicating with care teams. We explored the quantity and quality of caregiver participation during recorded clinical appointments in women with metastatic breast cancer.

**Methods:**

This was a convergent parallel mixed methods study. Caregiver participation quality was operationalized using a summative thematic content analysis to identify and sum caregiver roles performed during appointments. Caregiver participation quantity was measured by calculating the proportion of speaking time. Participation quality and quantity were compared to patient activation, assessed using the Patient Activation Measure.

**Results:**

Fifty-three clinical encounters were recorded. Identified caregiver roles included: General Support; Management of Treatment or Medication; Treatment History; Decision-Making; Insurance or Money; Pharmacy; Scheduling; Travel Concerns; General Cancer Understanding; Patient Specific Cancer Understanding; Caregiver-Initiated or Emphasis on Symptom Severity; and Caregiver Back-Up of Patient Symptom Description. Caregivers averaged 5 roles (SD 3): 48% of patients had low quality (< 5 roles) and 52% had high quality (> 6 roles). Regarding quantity, caregivers spoke on average for 4% of the encounter, with 60% of patients having low quantity (< 4%) and 40% of patients having high quantity (> 4%). Greater quality and quantity of caregiver participation was associated with greater patient activation.

**Conclusions:**

Caregivers perform a variety of roles during oncological decision-making visits aiding both patient and provider. Greater participation in terms of quantity and quality by the caregiver was associated with greater patient activism, indicating a need for better integration of the caregiver in clinical decision-making environments.

## INTRODUCTION

Caregivers constitute a pivotal role in assisting with the health care decision-making of individuals undergoing treatment for cancer. Caregiver roles are wide-ranging, including assisting with navigating health care systems, decisions about starting and stopping treatment, the treatment plan, and where to get treatment.^1,2^ These roles may be more extensive and dynamic in advanced illness conditions such as metastatic breast cancer (MBC), as the longer disease course and repeated instances of treatment changes result in multiple opportunities for care-related decisions.^3,4^ Beyond the type, timing, and location of treatment, decisions also include logistical and financial concerns, attending appointments, and getting access to treatment.^5–7^

The impact of cancer caregivers’ roles in patients’ decision-making can be both positive and negative. In a nationwide sample of over 5,200 newly diagnosed cancer patients, nearly half indicated that they shared treatment decision-making responsibility equally with a partner or another family member, and one in five solicited insight from someone close to them.^4,8^ Caregiver involvement has also been associated with increased treatment satisfaction and adherence.^5,7,9–12^ Researchers have found caregiver involvement to be highly influential on patient self-management behaviors, self-efficacy, stress, and depression.^13–15^ Additionally, caregiver health literacy affects treatment outcomes and self-care behaviors in patients with cancer, and they may employ this comprehension of health information and services when making decisions.^16,17^ Caregivers are often individuals of high importance and trust to the patient, meaning that they may have unparalleled insight into the patient’s life and can champion problems and preferences expressed by the patient outside of the clinical setting.^18^ At the same time, greater involvement of the caregiver in the clinical encounter could have negative consequences. Caregivers may overshadow the patient and heighten their distress by pushing for treatment options that are inconsistent with the patient’s personal values.^4,8,19,20^ Laidsaar-Powell and colleagues highlight this variability in the caregivers’ interaction in clinical encounters as variable and ranging from “active partner” to “welcome guest” to “intruder”. ^4^

Little is known about the range of caregivers’ roles during clinical encounters and how these may affect patient activation -- defined as the skills, knowledge, and confidence to manage one’s own health as an active participant.^21^ Higher patient activation has been associated with positive care experiences, uptake of self-management behaviors, and improved outcomes.^22^ Because caregivers often play extensive roles in the patient’s cancer trajectory, it is crucial to delve into the impact of caregiver interactions on patient engagement during clinical encounters. Therefore, this study sought to characterize caregiver involvement in and its correlation with patient activation during treatment decision-making visits for women with metastatic breast cancer.

## METHODS

### Study design and sample

We conducted a mixed-methods analysis using a convergent parallel design to explore the effect of caregiver participation in clinical encounters in women with MBC. This constituted a sub-study within a larger randomized control trial (RCT) evaluating the impact of formalized shared treatment decision-making planning on women with metastatic breast cancer (NCT 03806738). Patients participating in the parent RCT were asked for additional permission to audio-record their treatment decision-making encounter with their oncologist. Patients eligible for this sub-study included women aged 18 and older who were diagnosed with and receiving treatment for MBC at the University of Alabama at Birmingham (UAB). Only patients who were accompanied by a caregiver to the recorded appointment were included in this analysis. Demographic and clinical data was extracted from the parent RCT, including age, race, and home address (utilized to calculate distance travelled to clinic). This study complies with the Declaration of Helsinki was approved by the UAB Institutional Review Board (IRB-300002283).

### Qualitative data: Caregiver Roles and Quality of Participation

Treatment decision-making encounter recordings were transcribed by an independent transcription service and verified by the study team. To facilitate the exploration of the triadic relationship between the oncologist, the patient, and the caregiver, Wolff and Roter^23^ posited the family involvement in interpersonal health processes model that emphasizes relational rapport, information exchange, decision making, and goal setting. We utilized this framework to conduct a focused analysis of the triadic communication that occurs during treatment decision-making visits for women with metastatic breast cancer. Two independent coders (NH, GB) performed a summative content analysis,^25^ coding each verbal contribution of the caregiver according to the specific type of support that they were providing to the patient/oncologist using NVivo software. After the first round of open coding, coders worked in conjunction with the Principal Investigator (GR) to identify major and minor roles to add to the formal codebook of caregiver participation quality.

We then utilized these qualitative insights to operationalize and quantitatively measure the quality of caregiver participation,^23^ defined in this study as the number of supportive roles the caregiver plays during each decision-making encounter. Thus, caregiver participation quality was calculated by summing each of the role types performed by the caregiver during the recorded appointment. Each coder summed roles independently for each participant, facilitating the use of inter-coder correlations to establish inter-coder reliability. The correlation between the two coders’ computation of the quality measure was quite strong (r (58) = 0.989, p < 0.001), indicating robust interrater reliability. Finally, the sample was dichotomized at the mean to identify those patients with “low caregiver participation quality” and “high caregiver participation quality.” This enabled a direct comparison between the effects of the quality and quantity of caregiver participation on patient activation.

### Quantitative data: Quantity of Caregiver Participation

Quantity of caregiver participation was operationalized through timecoding of the recorded appointments. The recording began when the oncologist entered the room and stopped the recording once their encounter with the patient was complete. In each appointment, the treatment decision-point was defined as the time when the patient and provider reached consensus on the next step in the individual’s treatment plan. In some cases, an oncology fellow, pharmacist, or other healthcare professional was involved in the conversation, but these sections were only included in the time analysis if they occurred before the treatment decision. For example, oncology fellows often met with patients prior to the oncologist to ascertain their medical history and current status. The fellows would then relay this information to the oncologist, thereby shortening the amount of time that the oncologist needed to spend in the appointment. Conversely, timing of pharmacists in the encounters was variable. Some pharmacists discussed treatment options prior to the treatment decision point, while others entered the conversation once a definitive plan had been established. In these latter cases, the patient’s discussion with the pharmacist was excluded. Measured time-related variables included total appointment time, as well as total speaking time for the patient, caregiver, oncologist, and other health professionals. Proportion of speaking time was then calculated by dividing each individual’s speaking time by the total appointment time. Again, caregiver speaking time was dichotomized at the mean to identify patients with “low caregiver participation quantity” and “high caregiver participation quantity.”

### Integration: Effect of Quantity and Quality of Caregiver Participation on Patient Activation

Descriptive statistics, including frequencies, means, standard deviations (SDs), and ranges, for caregiver quality and quantity of participation were calculated. The dichotomized caregiver quality and quantity of participation measures were cross-tabulated to identify proportions of both quantity and quality of caregiver participation. This resulted in four subgroups: low quality/low quantity, low quality/high quantity, high quality/low quantity, and high quality/high quantity.

The patient’s level of engagement in their healthcare was assessed using the Patient Activation Measure (PAM), a 13-item questionnaire that assesses patients’ knowledge, skill, and confidence in managing their own health and healthcare. PAM is scored 1–100, with higher scores representing higher patient activation. PAM was measured 1-month post-treatment decision.^21^ Caregiver participation quality and quantity were then compared to patient activation levels independently through t-tests and in combination through an error bar chart.

## RESULTS

### Sample characteristics

In the sixty recorded appointments, fifty-three patients (88%) were accompanied by a caregiver to their appointment. Demographic information for these patients is available in Table 1. Patients with caregivers were a mean of 57 years old (SD 11) and most often White (70%). Almost all patients were diagnosed with recurrent MBC (90%), and 47% traveled over an hour to receive care at UAB. Demographic information was not collected for caregivers, but 35% of caregivers were verbally identified as the patient’s spouse or partner, 27% were identified as friends, sisters, daughters, or other family members, and 38% of caregivers were not identified relationally during appointments.

### Caregiver Roles

The majority of caregivers (85%) participated in the treatment decision-making conversation at least once. Twelve specific minor roles were performed by caregivers in the sample within the larger themes of caretaking (79%), treatment decision-making (70%), logistics (64%), cancer understanding (55%), and symptom discussion (55%; Table 2).

### Caretaking:

Nearly 80% of caregivers in the study provided patient-related background information or performed caretaking actions during the appointment. These statements took three forms: (1) General Support, (2) Management of Medication/Treatment, and (3) Treatment History Recall. Instances of general support were performed by 64% of the caregivers and included statements demonstrating that the caregiver supports the patient as an individual person, both inside and outside of their treatment management. For example, caregivers described their relationship to the patient (“I’m X’s husband”), provided encouragement, or discussed shared responsibilities at home (e.g., cleaning, cooking meals). Caregivers also contributed considerably to discussions of medical caretaking, with over 60% asking questions related to current or future medications or treatment and over 40% aiding in the recall of the patient’s treatment history. Management questions revolved around frequency, amount, and timeline of treatments. Caregivers also aided in the recall of treatment history by providing names and timelines of previous medications, treatment plans, or providers.

### Decision-making:

The second major component of caregiver advocacy was their contributions to the treatment decision-making process (70%; 4). Often, these inputs were questions about potential treatment options, including how they work, likelihood of success, their side effects, and the provider’s opinion about the best choice for the patient. Questions regarding accessibility or logistics of new medications were also included here if they were regarding the comparison of one potential treatment to another. Finally, explicit encouragement towards specific treatments and discussions of advanced directives/power of attorney were also included under the decision-making role.

### Logistics:

64% of caregivers contributed to discussions regarding logistics making it the third major component. These discussions included the specific roles of (5) appointment scheduling (47%), (6) travel concerns (40%), (7) pharmacy (19%), and (8) insurance or finances (17%). Caregivers often helped patients recall when appointments with other members of their healthcare team were scheduled and offered input on future times that would potentially be good for themselves and/or the patient. Travel concerns then encapsulated any mention of the time/distance necessary for the patient to travel in order to receive care. It is important to mention that these statements often served two purposes, either to advocate for a more efficient use of the patient’s time by better organizing their schedule or by expressing their (the caregiver’s) willingness to travel greater distances or more often in order to better support the patient in their care. In either case, the caregivers were clearly aware of the travel burden and were working to alleviate its potential stress on the patient. Some caregivers also discussed pharmacy options, including questioning where specific medications would be filled or stating that they are involved in obtaining the medication for the patient. And, finally, caregivers would ask financial or insurance related questions that would often prompt the involvement of another care-team member than the oncologist.

### Cancer understanding:

The fourth most common role was related to cancer understanding, which was discussed by 58% of caregivers. These discussions were further subdivided into (9) questions/statements regarding the understanding of how cancer works in general (13%) versus (10) seeking clarification or more information about the patient’s cancer specifically (51%). In each of these incidences, the purpose of the caregiver’s participation was to improve either their own or the patient’s understanding of their diagnosis. These typically occurred in the beginning of the appointment when the oncologist was reviewing new and old scans and discussing why a treatment change was potentially necessary.

### Symptom discussion:

The final major role involved the discussion of patient symptoms and side-effects (55%). These contributions were subdivided into two categories based on whether the discussion was initiated by (11) the patient (38%) or (12) the caregiver (43%). In the former case, the caregiver acted as a back-up or support to the patient, confirming that the patient’s presentation of symptoms was accurate. In other cases, however, it was the caregiver that prompted the discussion, or they actively disagreed with the patient’s characterization of their wellbeing. In each of these cases, the caregiver argued that various symptoms/side effects were a *greater* burden to the patient and sought the oncologist’s opinion about what could be done to better manage the experience.

### Caregiver Participation Quality

Based on the roles identified from the summative content analysis, a range of 0 through 12 potential roles were displayed, where 0 represented no contributions and 12 represented more diverse participation during the decision-making encounter. The mean number of minor roles performed during the decision-making encounter was 5 (SD 3). Of caregivers present, eight (15%) did not perform any roles and only one performed every identified role during the clinical encounter. A slight majority of patients (52%) had caregivers with high quality participation, while 48% had caregivers with low quality participation.

### Caregiver Participation Quantity

Average appointment time for all patients was 29 minutes (SD 13), with oncologists speaking for an average 76% of the decision-making encounter. Patients were the next most common contributor, speaking an average of 20% of the total appointment time, while caregivers contributed an average of 4% of the conversation. A majority of patients (60%) had caregivers with low quality participation, while 40% had caregivers with high quantity participation.

### Effect of Quantity and Quality of Caregiver Participation on Patient Activation

When combining caregiver participation quality and quantity (Table 3), the largest subgroup of patients (42%) had caregivers with both low quality and quantity of participation, 34% had both high quality and quantity of participation, 18% had high quality and low quantity of participation, and 6% had low quality and high quantity of participation.

**Figure 1:**
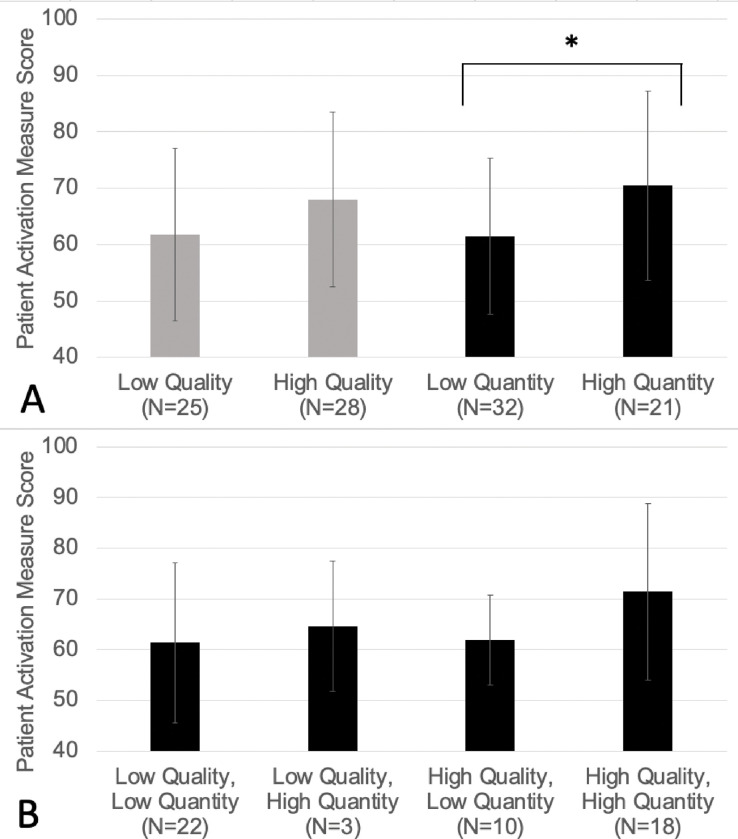
A and B: Means and Standard Deviations of Patient Activation by Quality and Quantity of Caregiver Participation (N=53)

Most patients (82%) reported a moderate to high level of activation (PAM score range 42–100, mean 65, SD 16). Patients with both low and high quality of caregiver participation had similar levels of patient activation (low: PAM score 62, SD 15; high: PAM score 68, SD 16; p = .07). Patients with high quantity of caregiver participation had slightly higher levels of patient activation when compared to those with low quantity of caregiver participation (high: PAM score 71, SD 17; low: PAM score 62, SD 14; p = .02; Fig. 1A). When examining the combined association of both quality and quantity of caregiver participation on patient activation (Fig. 1B), patients with both low quality and quantity of caregiver participation had the lowest mean PAM score (61, SD 16), while patients with both high quality and quantity of caregiver participation had the highest mean PAM score (71, SD 17). However, this difference was not statistically significant [F(3,49) = 1.625, p = 0.196].

## DISCUSSION

This study demonstrated that the quality and quantity of caregiver participation during decision-making visits for women with MBC positively impacted patient activation. We utilized content analysis and a constant comparative method to operationalize the quality of caregiver participation, defined as the number of minor roles performed by the caregiver during the appointment. These roles were subdivided into five major categories and included: General Support; Management of Treatment or Medication; Treatment History; Decision-Making; Insurance or Money; Pharmacy; Scheduling; Travel Concerns; General Cancer Understanding; Patient Specific Cancer Understanding; Caregiver-Initiated or Emphasis on Symptom Severity; and Caregiver Back-Up of Patient Symptom Description. This enabled us to capture multiple aspects and levels of caregiver support, ranging from their mere presence to their participation in conversation, to the tangible performance of patient advocacy and aid during the clinical encounter.

Among caregivers, quantity of conversation participation seemed to impact the quality of caregiver participation, in that the more they spoke, the more likely they were to perform diverse roles. However, this association was not perfectly consistent. Some caregivers were quite talkative but primarily discussed clinically irrelevant topics or did not keep the focus on the patient themself. Others rarely spoke during the appointment, but when they did, it was to offer clinically meaningful information. This interaction between quality and quantity highlights the importance of delving into the content of appointment discussions, as the more ways that the caregiver productively participates in the clinical encounter, the more empowered the patient is and the more comfortable and knowledgeable they feel in their treatment.

This finding adds to the emerging literature on the diverse characteristics of caregivers’ involvement and influence on patients.^1,4,24^ According to Acquati and colleagues, low caregiver involvement affects a patient’s adherence to and persistence with the treatment regime, irrespective of patient activation.^24^ The level of caregiver involvement is further extended to influencing patient’s decision-making, adherence to treatment, and practice of healthful behaviors.^25–27^ Furthermore, research on identity and relational needs in clinical settings educates this finding on caregiver contributions to appointment discussions and the patient decision-making process. Both Krieger and colleagues and Venetis and colleagues highlight the sensitivity to the caregiver and patient illness identity and relationship needs that influence caregiver involvement in clinical settings.^28,29^ This suggests focusing on interventions that address triadic interaction that includes caregiver participation in clinical settings.^4,29^

### Clinical Implications

Caregivers not only provide tangible and useful clinical information for healthcare providers during appointments, but they act as support systems and advocates for the patients. Caregivers have a unique perspective on the patient’s health and needs and can provide valuable information to healthcare providers that could increase their quality and satisfaction of care. Greater integration of the caregiver into decision-making conversations can help to ensure that the patient’s preferences and values are considered when developing a treatment plan and can improve communication and collaboration between healthcare providers and the patient’s support system. Physician recognition of the importance of these roles and movement towards greater integration of the caregiver into clinical encounters could therefore facilitate better understanding, agency, and satisfaction with the patient’s treatment experience.

### Limitations

Limitations for this study are similar to other qualitative and mixed methods studies in that the sample was relatively small and convenient. The sixty recorded appointments were less than half of the initial one-hundred twenty-six patients who consented to appointment recording; however, logistical challenges including visit conversion to telehealth during the COVID-19 pandemic and physicians not remembering to record limited the sample. Data was also collected from a single institution during a limited timeframe, which means that the sample may be non-representative of other physician, patient, and caregiver populations. Another limitation is that patient-caregiver relationships outside of the clinical context were not assessed and the strength of this relationship could impact the influence of the caregiver on patient activation and participation.

## Conclusions

Caregivers perform a variety of tasks and roles during oncological decision-making clinical visits that aid both the patient and the provider. Greater participation in terms of quantity and quality by the caregiver was associated with greater patient activism, indicating a need for better integration of the caregiver in the clinical decision-making environment. More research is needed regarding how best to incorporate caregivers while maintaining the patient’s preferences and agency in the clinical encounter.

## Figures and Tables

**Table 1:** Demographics of Patients and Presence of Caregivers in Appointments (N = 53)

	Total sample N (%)
**Caregiver Identity:** Partner/Spouse	21 (35%)
Other Identified*	16 (27%)
Unidentified	23 (38%)
**Age:** Under 50	14 (23%)
51–65	35 (58%)
66 and Older	11 (18%)
**Race:** White	42 (70%)
Black	18 (30%)
**Minutes Travelled to Appt:** Less than 30	14 (23%)
30– 1 Hour	13 (22%)
Over 1 Hour	28 (47%)

* included friends, siblings, and children of patient

**Table 2:** Components of Caregiver Activism during Clinical Decision-Making Encounters

Major Roles	Minor Roles	N	% (N = 53)
Caretaking		42	79
	General Support	34	64
	Management of Treatment or Medication	32	60
	Treatment History	23	43
Decision-Making		37	70
Logistics		34	64
	Insurance or Money	9	17
	Pharmacy	10	19
	Scheduling	25	47
	Travel Concerns	21	40
Cancer Understanding		29	55
	General Cancer Understanding	7	13
	Patient Specific Cancer Understanding	27	51
Symptom Discussion		29	55
	Caregiver-Initiated or Emphasis on Symptom Severity	23	43
	Back-up of Patient Symptom Description	20	38

**Table 3:** Crosstabulation of Quality and Quantity of Caregiver Participation

		Quantity of Caregiver Participation	
		Low	High
Quality of Caregiver Participation	Low	42% (22 Patients)	6% (3 Patients)
	High	18% (10 Patients)	34% (18 Patients)
